# Successful use of rivaroxaban in postoperative deep vein thrombosis of the lower limb following instability with warfarin: a case report

**DOI:** 10.1186/s13256-016-1058-5

**Published:** 2016-10-05

**Authors:** Mario Schiavoni, Antonella Coluccia

**Affiliations:** Department of Internal Medicine Hospital, Hemophilia and Rare Coagulopathies Centre, “I. Veris Delli Ponti”, 73020 Scorrano, Azienda Sanitaria Locale di Lecce Italy

**Keywords:** Rivaroxaban, Warfarin, Postoperative deep venous thrombosis, Case report

## Abstract

**Background:**

Evidence from clinical trials shows rivaroxaban to be effective for the treatment of deep vein thrombosis. Switching to rivaroxaban following failure of indirect anticoagulants in deep vein thrombosis has not been demonstrated in a real-life setting.

**Case presentation:**

A 43-year-old white woman was switched from warfarin to rivaroxaban for the treatment of thrombosis of her right common femoral vein after saphenectomy. The reason for the switch was due to the instability of anti-coagulation therapy with vitamin K antagonists over a period of 3 months during which she did not reach the “therapeutic range” of prothrombin time-international normalized ratio.

The ineffectiveness of the conventional oral anticoagulant was confirmed by persistence of moderate-high values of fibrin D dimers (780 ng/ml) and by residual vein thrombosis at an ultrasound examination. Objectively, her right leg appeared to be still edematous and warm and pain was elicited by deep palpation. Rivaroxaban was administered after warfarin discontinuation (prothrombin time-international normalized ratio = 1.43) at a dosage of 15 mg every 12 hours for 3 weeks, followed by 20 mg once daily for 3 months. After this period, her objective symptoms significantly improved, with reduction of edema of her lower limb and pain relief. Her fibrin D dimer values returned to normal (210 ng/ml). An ultrasound showed recanalization of the obstructed venous segment.

**Conclusions:**

In this case report, a switch to rivaroxaban from warfarin was shown to be effective and safe for the treatment of postoperative deep vein thrombosis, whereas standard oral anticoagulation therapy, which required dose adjustments over a period of 3 months, was not able to stabilize the therapeutic range of prothrombin time-international normalized ratio nor improve our patient’s outcome.

## Background

Anticoagulant therapy is effective for the treatment of acute venous thromboembolism (VTE): deep vein thrombosis (DVT) and pulmonary embolism (PE) [1]. For more than half a century, standard anticoagulant therapy with heparin (mainly subcutaneous low molecular weight heparin; LMWH) overlapped and followed by oral vitamin K antagonists (VKAs), such as warfarin, represented the mainstay recommended by guidelines worldwide [2]. The combination of LMWH/VKAs given for ≥3 months is capable of significantly reducing morbidity and mortality, preventing recurrent thromboembolism (RTE) during the acute phase and avoiding long-term complications such as post-thrombotic syndrome (PTS) and secondary pulmonary hypertension (SPH) after PE [3]. However, one of the major limitations of oral VKAs is the need for laboratory monitoring by prothrombin time test expressed as the international normalized ratio (PT-INR). In fact, this parameter must be kept in a “therapeutic range” of 2 to 3 (target 2.5) throughout the entire course of therapy (that is, time in therapeutic range; TTR), and this goal is achieved by dose adjustments. Unfortunately, it is not always possible to obtain optimal values of PT-INR due to several variables that may interfere with VKAs, such as concomitant medications and some foods, making the anticoagulant effect unstable and therefore increasing the risk of failure.

Recently, novel drugs termed “direct oral anticoagulants” (DOACs) have been introduced in clinical practice, including dabigatran, rivaroxaban, apixaban, and edoxaban, that were shown to be effective for the treatment of VTE [4–7], and represented an alternative to VKAs (defined as “indirect oral anticoagulants” [8]). DOACs have demonstrated the same efficacy and safety as standard therapy with combined LMWH/warfarin, but with the advantage of not requiring any laboratory monitoring with dose adjustments, due to a stable anticoagulant effect. In particular, in two clinical studies conducted in the setting of VTE (proximal DVT and PE) [5, 6], the efficacy of rivaroxaban was shown to be not inferior to that of conventional anticoagulant therapy. In a pooled analysis of the two studies, the drug also yielded a similar incidence of the main safety outcome but a significant reduction in the incidence of major bleeding: hazard ratio 0.54; 95 % confidence interval (CI) 0.37 to 0.79; *p* = 0.002 [9]. In randomized clinical trials, rivaroxaban was used for the initial treatment of an acute thrombotic event with a high dose (15 mg twice daily) administered for 3 weeks followed by a standard monodose (20 mg once daily) without the support of LMWH [5, 6]. Yet, in the real-life setting of DVT, no information is available on the switch to rivaroxaban after clinical failure with warfarin.

## Case presentation

**Fig. 1:**
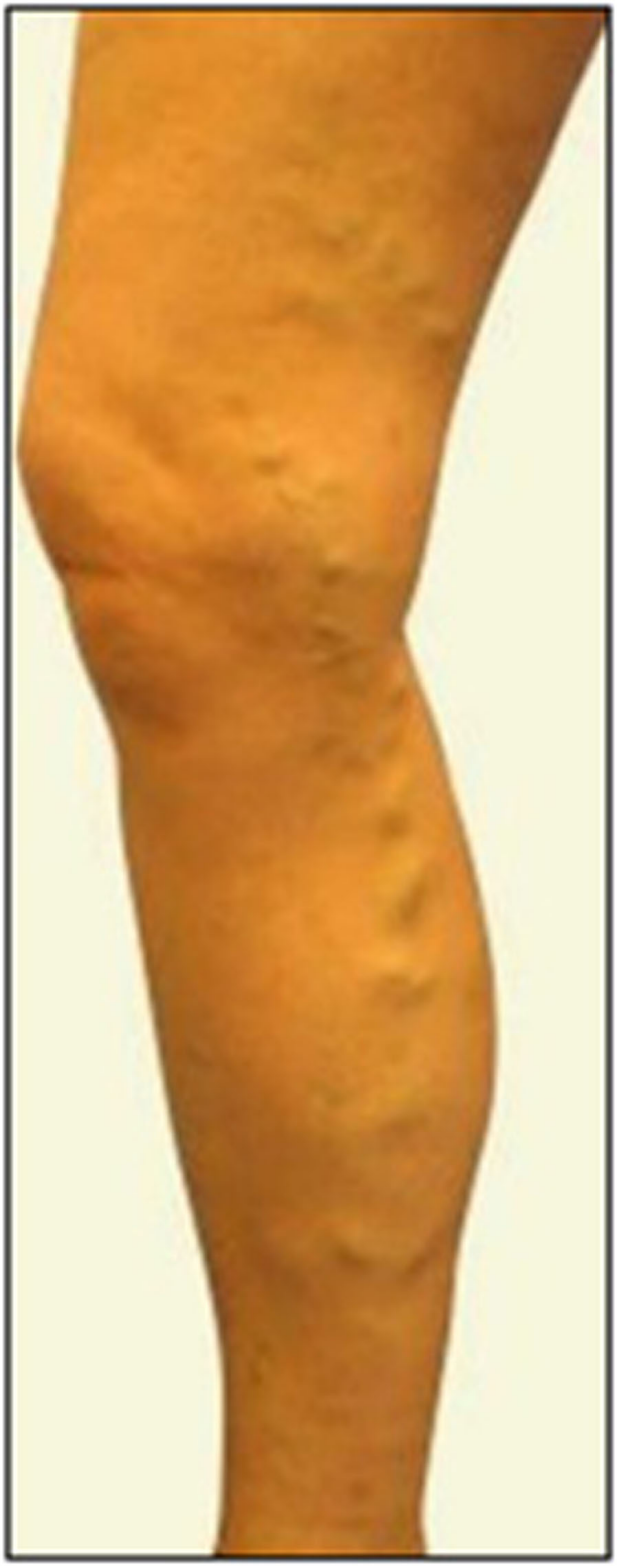
Varicose syndrome of right lower limb

A 43-year-old white woman underwent saphenectomy (Table 1) of her right lower limb because of severe venous insufficiency caused by varicose syndrome (Fig. 1). An ultrasound examination of superficial and deep veins of her lower limbs carried out before surgery was judged normal. The surgical intervention of “stripping” was performed using traditional techniques, with no apparent perioperative complications. Her postoperative course appeared normal.

**Table 1:** Intervention timeline

Intervention	Time point
Saphenectomy	Day 0
Edema due to postoperative proximal DVT; start of anticoagulant therapy (enoxaparin and warfarin)	Day 4 Up to 3 months
Therapy with rivaroxaban	Month 4 to 6

*DVT* deep vein thrombosis

### Postoperative deep venous thrombosis

**Fig. 2:**
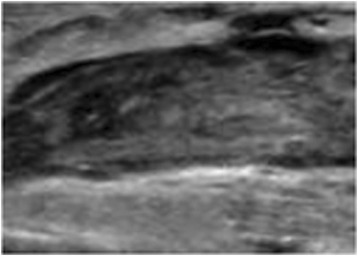
Ultrasound image of thrombosis of right common femoral vein

Despite all precautions, including early mobilization, on the fourth postoperative day (Table 1) she experienced a significant edema of her right lower limb, which appeared warm and tense. Subjectively, an acute and persistent pain was referred. Laboratory tests showed a moderate increase in leukocytes (15.7 × 10^3^/μl, reference values up to 10 × 10^3^/μl) and elevated levels of fibrin D dimers (1800 ng/ml; reference values up to 250 ng/ml). An ultrasound examination (Philips HD7; 7.5 MHz linear array transducer) revealed the presence of thrombosis of her common femoral vein in her operated limb (Fig. 2). Diagnosis of postoperative proximal DVT was made (Table 1).

### Anticoagulant therapy

Anticoagulant therapy with enoxaparin at a dosage of 100 IU/Kg subcutaneously every 12 hours was started overlapped with warfarin at adjusted dosage (Table 1). Heparin treatment was stopped when her PT-INR reached values between 2.0 and 2.5. She was discharged from our hospital with the indication to continue oral anticoagulants over a period of 3 months and to perform periodic laboratory monitoring (therapeutic range of PT-INR 2.0 to 3.0, target 2.5) initially every week (until optimal PT-INR) and every 3 weeks afterwards. An appropriate compression by elastic stockings was also prescribed [10].

### Instability of conventional anticoagulant therapy

**Fig. 3:**
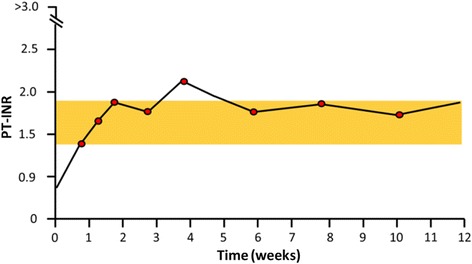
Instability of prothrombin time-international normalized ratio monitoring during the 3-month period of therapy with warfarin. *Orange* highlighted area shows instability range

Laboratory tests were periodically performed in order to monitor the efficacy of oral anticoagulant therapy with warfarin. Despite frequent dose adjustments, values of PT-INR in the “therapeutic” range were reached only once (Fig. 3).

**Fig. 4:**
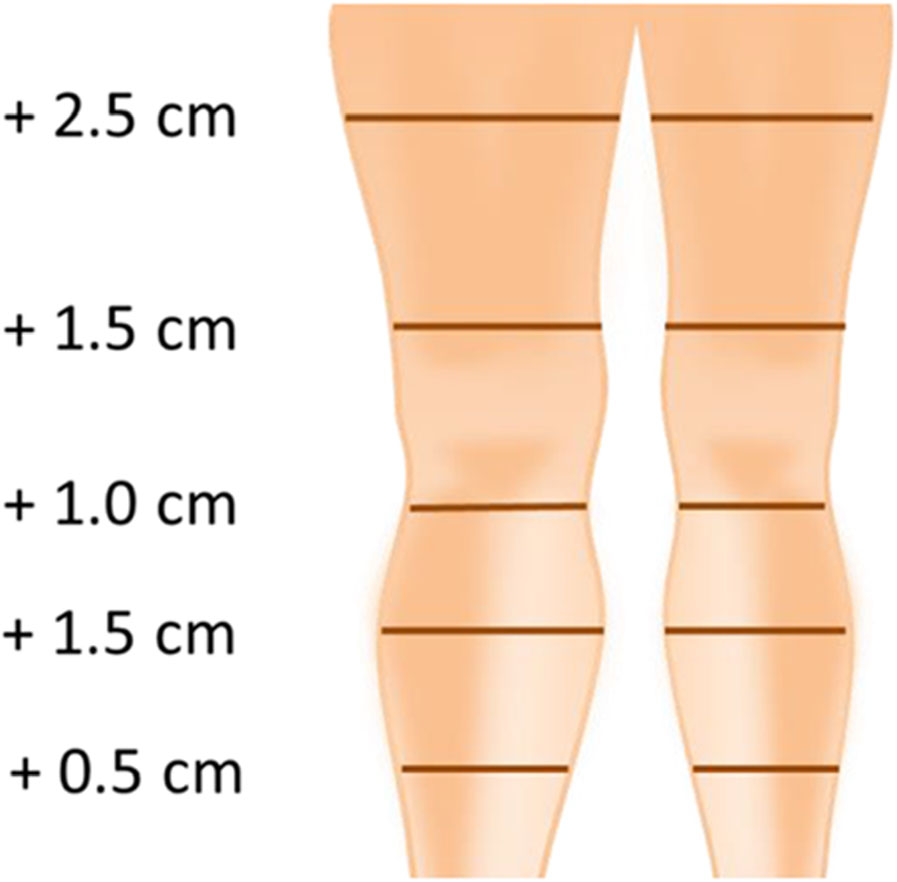
Swelling of the right leg compared with left leg after 3-month period of therapy with warfarin

**Fig. 5:**
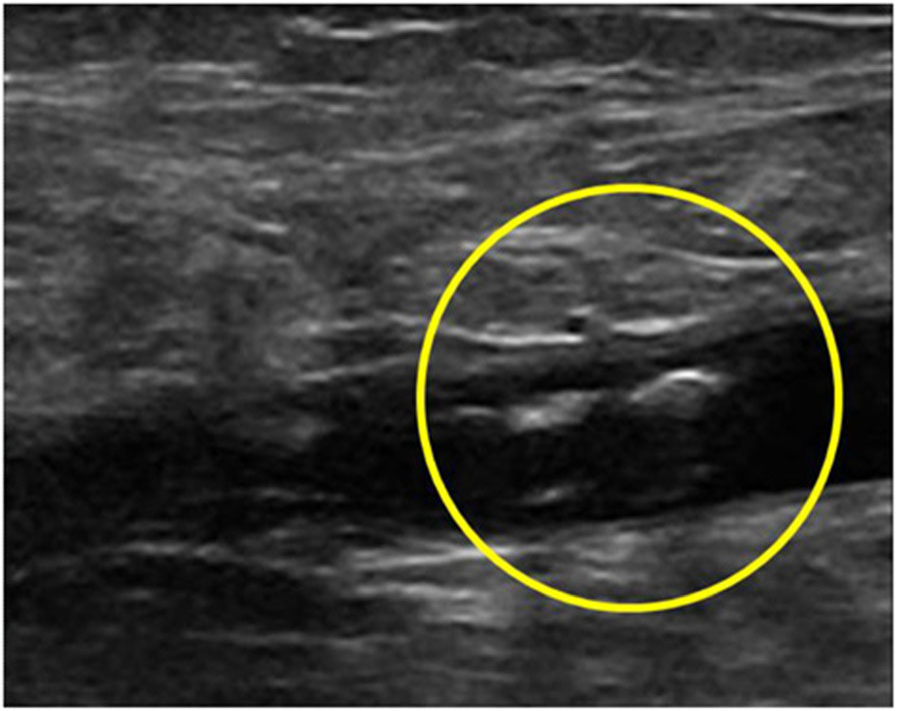
Residual thrombosis (circle) of right common femoral vein after 3-month period of therapy with warfarin

She subjectively presented heaviness and aching of her right lower limb, which appeared objectively still hot and swollen with an increased circumference of 0.5 to 2.5 cm in comparison with her left leg (Fig. 4). Her values of fibrin D dimers were moderately high (780 ng/ml) and a residual vein thrombosis was shown by the echoDoppler (Fig. 5).

### Switch to rivaroxaban

Because of the instability of conventional oral anticoagulants and the poor clinical outcome, warfarin was discontinued when her value of PT-INR was 1.43. Rivaroxaban was started at an intensive dosage of 15 mg every 12 hours for 3 weeks, followed by 20 mg/day for 3 months (Table 1).

### Patient outcome

**Fig. 6:**
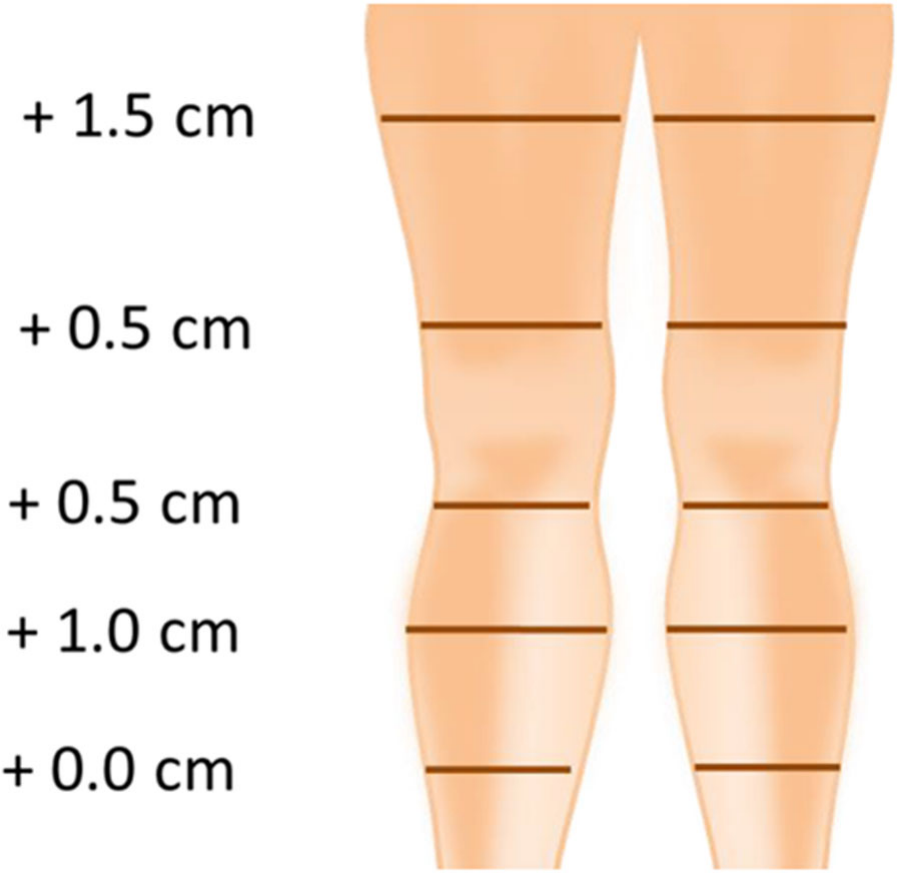
The right leg compared with the left leg after 3-month therapy with rivaroxaban

**Fig. 7:**
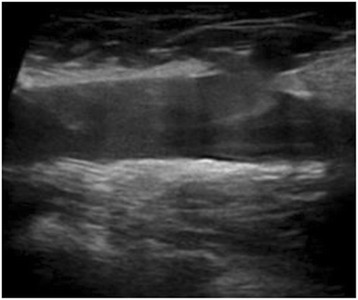
Ultrasound image of the complete resolution of the right common femoral vein thrombosis after 3-month therapy with rivaroxaban

Three months after the induction of rivaroxaban, she showed a significant improvement in symptoms, with an objective evident reduction of edema of her right leg (Fig. 6), as well as a subjective pain relief with recovery of walking without interruption. Laboratory monitoring showed a return to a normal leukocyte count (8.7 × 10^3^/μl) and fibrin D dimers values (210 ng/ml). An ultrasound examination confirmed the complete resolution of DVT (Fig. 7).

## Discussion

VTE including DVT and PE is a serious clinical condition that requires anticoagulants as first-line treatment [11]. Currently, conventional therapy consists of LMWH followed by VKAs. Although this approach is effective, it has some limitations, such as the need for frequent laboratory monitoring and, often, difficulty in reaching the therapeutic range of the PT-INR with consequent instable anticoagulant effect and poor clinical outcome. The inadequate management of VTE by standard VKAs may cause RTE and long-term serious sequelae including PTS and chronic SPH in case of PE, which represent a further burden for these patients [11].

When used as monotherapy for VTE, rivaroxaban showed similar efficacy and safety compared to the current standard therapy, but without the need for routine laboratory monitoring [9, 11]. In our patient, the instability of conventional anticoagulation therapy with combined LMWH/warfarin as well as the persistence of objective and subjective symptoms after 3 months of treatment led us to switch from warfarin to rivaroxaban for a period of 3 more months. Oral rivaroxaban was able to induce complete resolution of her venous thrombosis, as shown by clinical, laboratory, and ultrasound imaging results. Neither bleedings nor renal venous thrombosis (RVT) was observed during her entire course of treatment. Conversely, the conventional adjusted therapy with LMWH/warfarin could not stabilize her PT-INR and did not improve her clinical outcome.

Our patient was switched to rivaroxaban after discontinuation of warfarin, when her PT-INR value was 1.43, according to recent recommendations, although not evidence based [12]. In fact, to the best of our knowledge, this is the first example of switching to rivaroxaban in the treatment of DVT. In fact, this strategy has been investigated but only in healthy individuals and with regard to the pharmacodynamics and pharmacokinetics during the transition from warfarin to rivaroxaban [13].

## Conclusions

Given the positive outcome, our approach may be considered in similar clinical situations. In our experience, switching to rivaroxaban from conventional combined therapy with LMWH/warfarin was effective and easily manageable. Neither undesirable side effects nor complications such as RTE and bleeding occurred throughout the treatment period. Worth noting, the simple once-daily oral administration of rivaroxaban improved patient adherence to the extended period of anticoagulant therapy.

## Abbreviations

CI: Confidence interval
DOACs: Direct oral anticoagulants
DVT: Deep vein thrombosis
LMWH: Low molecular weight heparin
PE: Pulmonary embolism
PT-INR: Prothrombin time test expressed as the international normalized ratio
PTS: Post-thrombotic syndrome
RTE: Recurrent thromboembolism
RVT: Renal venous thrombosis
SPH: Secondary pulmonary hypertension
TTR: Time in therapeutic range
VKAs: Vitamin K antagonists
VTE: Venous thromboembolism
